# General and abdominal obesity prevelances and their relations with metabolic syndrome components

**DOI:** 10.12669/pjms.35.4.235

**Published:** 2019

**Authors:** Olgun Goktas, Canan Ersoy, Ilker Ercan, Fatma Ezgi Can

**Affiliations:** 1 Dr. Olgun Goktas, Associate Professor, Family Health Center, Nilufer, Uludag University, Bursa, Turkey; 2 Prof. Dr. Canan Ersoy, Department of Internal Medicine, Division of Endocrinology and Metabolism, Faculty of Medicine, Uludag University, Bursa, Turkey; 3 Prof. Dr. Ilker Ercan, Department of Biostatistics, Faculty of Medicine, Uludag University, Bursa, Turkey; 4 Fatma Ezgi Can, Bsc Department of Biostatistics, Institute of Health Science, Uludag University, Bursa, Turkey

**Keywords:** Abdominal obesity, Obesity, Metabolic syndrome

## Abstract

**Objective:**

To evaluate the frequency of obesity and its relation of metabolic syndrome.

**Methods:**

The data from the records of the consecutive adult residents of Bursa province in Turkey who were admitted to the family health centers from the 1^st^ January to the 31^st^ December 2016 were evaluated retrospectively. The population size was 2901396 (N) and the sample size was at least n=17729. A total of 17812 participants (10939 females, 6873 males) were included in this retrospective observational study. Sociodemographic characteristics, diseases, used medication and smoking, height, weight, waist and hip circumferences (WaC and HC) were recorded from the files.

**Results:**

The mean age of all subjects was 46.1 years, the mean BMI was 28.1 kg/m^2^ with a mean WaC of 91.3 cm, HC of 104.7 cm, WHR of 0.87. The prevalence of obesity in Bursa was found to be 32.2% (37.8% in females and 23.3% in males) according to BMI, 63.2% (69.7% in females, 52.9% in males) according to waist and hip circumferences.

**Conclusions:**

Preventive measures should be taken by health authorities to prevent the rapid increase in general and abdominal obesity that may lead to serious comorbidities.

## INTRODUCTION

Obesity is an important health problem affecting individual and society all over the world, as well as in our country, due to its association with increased morbidity and mortality. Other than dietary and physical activity habits, there are many factors affecting body weight increment like age, gender, marital status, education level, accompanying illnesses and drugs used.^1^,^2^

Assessment of obesity is mostly made by measuring body mass index (BMI) which is a parameter of general obesity. Beside this, obesity is also assessed by measurement of waist and hip circumferences (WaC, HC) and calculation of waist to hip ratio (WHR).^3^,^4^

Abdominal obesity has much more health risk according to the fat excess in other regions of the body. Evaluation of optimal WaC is made differently by organizations like International Diabetes Federation (IDF) and World Health Organization (WHO). WaC and WHR are closely related to metabolic syndrome components which are abdominal obesity, insulin resistance and related disorders. Metabolic syndrome includes the components such as abdominal obesity, glucose intolerance, dyslipidemia, hypertension and atherosclerotic heart disease. Each component has harmful effects on human health, morbidity and mortality.^4^-^8^

There are different national studies conducted in our country evaluating obesity prevelance in adults from 1990 and 2010. In these studies obesity prevelance ranges between 18.8% to 39.7% with an increasing rate in recent years.^2^,^9^ In a previous study conductedby us and reported in 2005, obesity prevelance was found to be 35.5% in our city Bursa and we wanted to check this prevalence.^10^

## METHODS

In the study, the population size of Bursa province (N=2901396) was accepted as the main population and the sample size was at least n=17729 at the level of significance of d=0.009 and at the level of significance of α=0.01 with reference to 36% obesity incidence rate.^9^ Sampling was based on size proportional stratified sampling. A total of 26356 individuals from 19 databases of family health centers identified by stratification were included in the study. When the subjects with missing data were excluded from the study, a total of 17812 participants were included.

Age, gender, marital status, educational status, accompanying diseases, medications used, height, weight, WaC and HC were recorded from the files. BMI and WHR were calculated. BMI was computed by dividing body weight in kilograms to height in meters squared. The WHR was calculated by the division of WaC to HC as an index of abdominal obesity.^3^,^4^ The prevalences of obesity in our study were assessed according to BMI, WaC (IDF and WHO criterias) and WHR. Obesity classification according to BMI was ≥30 kg/m² for both genders, according to WaC defined by IDF was ≥94 cm for males and ≥80 cm for females and by WHO was ≥102 cm for males and ≥88 cm for females. According to WHR, subjects were considered as obese if >0.9 for males and >0.8 for females.^11^,^12^

The data of the consecutive adult residents of Bursa who were admitted to the family health centers due to routine control programmes from the 1^st^ January to the 31^st^ December 2016 were evaluated retrospectively. The data from the records of subjects older than 18 years of age were included in the study.

The necessary physical examinations and hormonal evaluations were performed to exclude endocrinological causes for obesity. Medication records were evaluated. Data of subjects with endocrine obesity and with usage of drugs that increase insulin resistance like steroids were not included in the study.

With reference to obesity evaluation according to BMI index, factors affecting the risk of obesity were investigated by backward binary logistic regression. Binary logistic regression analysis indicated regression coefficients, standard error of coefficients, odds ratio and 95% confidence intervals. The effect size was calculated for the mean values d=(μ_2_-μ_1_)/σ*pooled* for the ratios *h = φ_1_-φ_2_* and *φ_i_ = 2arcsin√P_i_*.^13^,^14^ Effect size value was evaluated according to 0.2 low, 0.5 moderate, 0.8 high, 1.3 very high.^15^ P<0.05 was considered statistically significant. The analysis was done in the IBM SPSS v.20 program (Uludağ University).

## RESULTS

Data from the files of a total of 17812 subjects (10939 females; 61.41%, 6873 males; 38,59%) were included in the study. The mean age of all subjects was 46.13±18.52 years, the mean BMI was 28.10±9.36 kg/m^2^ with a mean WaC of 91.33±14.74 cm, HC of 104.70±11.74 cm, WHR of 0.87±0.10 (Table-I).

**Table I:** The demographic features of both genders and all participants.

Variables	Females (n=10939)	Males (n=6873)	All participants (n=17812)
Age (years)	45.92±18.17	46.45±19.06	46.13±18.52
Height (cm)	158.05±18.17	172.63±10.72	163.67±17.25
Weight (kg)	72.61±15.12	80.43±14.47	75.63±15.35
BMI (kg/m2)	28.75±9.53	27.09±9	28.10±9.36
WaC (cm)	89.38±15.21	94.37±13.42	91.33±14.74
HC (cm)	106.13±12.52	102.46±9.99	104.70±11.74
WHR	0.84±0.09	0.92±0.09	0.87±0.10

BMI: body mass index, WaC: waist circumference, HC: hip circumference, WHR: waist to hip ratio

The prevalence of obesity in Bursa was found to be 32.2% (37.8% in females, 23.3% in males) according to BMI, 63.2% (69.7% in females, 52.9% in males) according to WaC-IDF criteria, 44.4% (53.6% in females, 29.8% in males) according to WaC-WHO criteria and 64.1% (67.8% in females, 58.4% in males) according to WHR (Table-II).

**Table II:** Obesity rates according to BMI, WaC and WHR criterias in both genders and all participants.

Variables, % (n)	Females (n=10939)	Males (n=6873)	All participants (n=17812)
BMI (kg/m2)	37.8 (4090/10806)	23.3 (1597/6857)	32.2 (5687/17663)
WaC-IDF (cm)	69.7 (5860/8402)	52.9 (2842/5372)	63.2 (8702/13774)
WaC-WHO (cm)	53.6 (4507/8402)	29.8 (1602/5372)	44.4 (6109/13774)
WHR	67.8 (5690/8391)	58.4 (3134/5366)	64.1 (8824/13757)

BMI: Body mass index, WaC: Waist circumference, WHR: Waist to hip ratio.

The risk of obesity increased 1.003 fold with increasing age. Female subjects were 1.976 times more likely to be obese compared to male subjects, married ones 1.609 times compared to singles and those having lower education level 1.812 times compared to higher education level subjects. The risk of obesity in those with previous obesity history was 6.326 times higher compared to those without, smokers 1.238 times compared to nonsmokers, hypertensives 1.921 fold compared to normotensives, dyslipidemics 1.217 fold compared to normolipidemics and hypothyroids 1.245 fold compared to euthyroids, diabetics with antidiabetic drug usage 1.624 times compared to nondiabetics (Table-III).

**Table III:** Investigation of risk factors affecting obesity (n=12505).

	β	SE of (β)	Exp (β)	95% CI		p value
Age	0.003	0.001	1.003	1.001	1.006	0.014
Gender (Ref: Male)	0.681	0.049	1.976	1.795	2.174	p<0.001
Marital Status (Ref: Single)	0.476	0.051	1.609	1.455	1.780	p<0.001
Education (Ref: High school and university)	0.595	0.052	1.812	1.635	2.008	p<0.001
Previous obesity history (Ref: No)	1.845	0.132	6.326	4.882	8.197	p<0.001
Smoking (Ref: No)	0.214	0.066	1.238	1.088	1.409	p<0.001
Hypertension (Ref: No)	0.653	0.056	1.921	1.721	2.144	p<0.001
Dyslipidemia (Ref: No)	0.196	0.069	1.217	1.064	1.392	0.004
Hypothyroidism (Ref: No)	0.219	0.108	1.245	1.007	1.539	0.043
Antidiabetic drugs(Ref: No)	0.485	0.056	1.624	1.454	1.814	p<0.001

* The regression model is statistically significant (p<0.001).

When the effective size of age showed a high effect on WaC-WHO in females and moderate effect in males. When the effect size of type 2 diabetes was evaluated it had low effect on WaC-IDF and WaC-WHO in males and moderate effect in females. Dyslipidemia was evaluated accoprding to the WaC-IDF in females which had a moderate effect. Atherosclerotic heart disease was found to have a low effect on all obesity parameters in both genders (Table-IV).

**Table IV:** Assessments of obese and non-obese female (a) and male (b) subjects according to effect size of their risk factors as metabolic syndrome components on general and abdominal obesity indexes.

a)				

		Female subjects		

Variables	Indexes	Obesity present	Obesity absent	ES±SD CI %95
Age	BMI	52.34±16.13 (51.84;52.83)	42.24±18.27 (41.81;42.68)	0.58±0.02 (0.538;0.617)
	WHR	51.09±17.825 (50.62;51.55)	37.12±14.153 (36.58;37.65)	0.83±0.024 (0.787;0.882)
	WaC-IDF	52.53±16.408 (52.11;52.95)	32.91±16.408 (32.39;33.42)	1.26±0.026 (1.212;1.312)
	WaC-WHO	55.01±15.59 (54.55;55.46)	36.86±15.45 (36.37;37.34)	1.17±0.024 (1.123;1.216)
Type 2 diabetes	BMI	0.26±0.007 (0.245;0.272)	0.12±0.004 (0.115;0.130)	0.35±0.02 (0.31;0.39)
	WHR	0.22±0.005 (0.208;0.23)	0.06±0.005 (0.05;0.067)	0.49±0.024 (0.441;0.534)
	WaC-IDF	0.23±0.005 (0.217;0.239)	0.03±0.003 (0.022;0.035)	0.65±0.024 (0.607;0.703)
	WaC-WHO	0.27±0.007 (0.255;0.281)	0.05±0.004 (0.045;0.059)	0.63±0.022 (0.586;0.674)
Hypertension	BMI	0.42±0.008 (0.404;0.434)	0.20±0.005 (0.191;0.21)	0.48±0.02 (0.44;0.52)
	WHR	0.38±0.006 (0.365;0.39)	0.14±0.007 (0.131;0.158)	0.54±0.024 (0.45;0.591)
	WaC-IDF	0.93±0.003 (0.923;0.937)	0.07±0.005 (0.061;0.081)	2.07±0.029 (2.011;2.123)
	WaC-WHO	0.46±0.007 (0.443;0.472)	0.12±0.005 (0.113;0.134)	0.77±0.023 (0.722;0.811)
Dyslipidemia	BMI	0.15±0.006 (0.14;0.162)	0.07±0.003 (0.064;0.076)	0.26±0.02 (0.22;0.30)
	WHR	0.14±0.005 (0.128;0.146)	0.041±0.004 (0.034;0.049)	0.35±0.024 (0.304;0.40)
	WaC-IDF	0.14±0.005 (0.135;0.153)	0.02±0.003 (0.014;0.025)	0.50±0.024 (0.453;0.548)
	WaC-WHO	0.17±0.006 (0.154;0.176)	0.04±0.003 (0.032;0.044)	0.44±0.022 (0.400;0.487)
Atherosclerotic heart disease	BMI	0.12±0.005 (0.107;0.127)	0.06±0.003 (0.05;0.061)	0.22±0.02 (0.18;0.26)
	WHR	0.11±0.004 (0.098;0.114)	0.03±0.003 (0.026;0.039)	0.30±0.023 (0.255;0.347)
	WaC-IDF	0.11±0.004 (0.102;0.118)	0.02±0.003 (0.012;0.022)	0.41±0.024 (0.365;0.459)
	WaC-WHO	0.13±0.005 (0.117;0.136)	0.03±0.003 (0.025;0.035)	0.38±0.022 (0.336;0.423)

b)				

		*Male subjects*		

*Variables*	*Indexes*	*Obesity (+) present*	*Obesity (-) absent*	*ES±SS SD CI %95*

Age	BMI	53.24±15.99 (52.45;54.02)	44.38±19.42 (43.86;44.91)	0.47±0.03 (0.42;0.53)
	WHR	53.02±16.725 (52.42;53.60)	37.10±18.356 (36.33;37.86)	0.91±0.029 (0.857;0.971)
	WaC-IDF	53.78±16.246 (53.18;54.37)	38.20±18.712 (37.47;38.93)	0.89±0.029 (0.837;0.949)
	WaC-WHO	55.12±15.389 (54.37;55.88)	42.75±19.334 (42.13;43.37)	0.68±0.031 (0.618;0.738)
Type 2 diabetes	BMI	0.23±0.011 (0.209;0.250)	0.15±0.005 (0.136;0.155)	0.22±0.03 (0.16;0.27)
	WHR	0.22±0.007 (0.203;0.232)	0.06±0.005 (0.047;0.066)	0.49±0.03 (0.44;0.55)
	WaC-IDF	0.22±0.008 (0.209;0.24)	0.07±0.005 (0.057;0.077)	0.46±0.028 (0.409;0.517)
	WaC-WHO	0.26±0.011 (0.24;0.283)	0.10±0.005 (0.093;0.113)	0.42±0.03 (0.36;0.479)
Hypertension	BMI	0.35±0.012 (0.322;0.368)	0.23±0.006 (0.220;0.243)	0.25±0.03 (0.20;0.31)
	WHR	0.38±0.009 (0.361;0.395)	0.11±0.007 (0.099;0.126)	0.64±0.03 (0.58;0.70)
	WaC-IDF	0.38±0.009 (0.358;0.394)	0.15±0.007 (0.13;0.16)	0.54±0.028 (0.481;0.590)
	WaC-WHO	0.42±0.012 (0.396;0.444)	0.20±0.007 (0.19;0.216)	0.48±0.03 (0.416;0.534)
Dyslipidemia	BMI	0.16±0.009 (0.138;0.174)	0.09±0.004 (0.079;0.094)	0.21±0.03 (0.16;0.27)
	WHR	0.16±0.006 (0.143;0.168)	0.04±0.004 (0.033;0.05)	0.40±0.03 (0.35;0.46)
	WaC-IDF	0.16±0.007 (0.144;0.17)	0.06±0.005 (0.05;0.069)	0.32±0.028 (0.267;0.375)
	WaC-WHO	0.18±0.01 (0.157;0.195)	0.08±0.004 (0.071;0.088)	0.29±0.03 (0.236;0.354)
Atherosclerotic heart disease	BMI	0.14±0.009 (0.122;0.156)	0.10±0.004 (0.089;0.105)	0.13±0.03 (0.08;0.19)
	WHR	0.16±0.006 (0.143;0.168)	0.05±0.005 (0.038;0.056)	0.37±0.03 (0.32;0.43)
	WaC-IDF	0.16±0.007 (0.143;0.170)	0.06±0.005 (0.05;0.069)	0.32±0.028 (0.265;0.373)
	WaC-WHO	0.17±0.009 (0.154;0.191)	0.08±0.005 (0.075;0.093)	0.27±0.03 (0.21;0.33)

Effect size (ES): 0.2 low, 0.5 moderate, 0.8 high, 1.3 very high.

BMI: body mass index, WaC: waist circumference, WHR: waist-to-hip ratio.

## DISCUSSION

Our study indicated high general and abdominal obesity in our population affecting the presence of components of metabolic syndrome. The prevelance of obesity increased as the cut off value for WaC was lowered as in WaC-IDF. Whatever criteria chosen (WHR, WaC-IDF and WaC-WHO) abdominal obesity seemed to be the prominent problem concerning metabolic syndrome components in both genders but especially in female gender. According to these results our hypothesis has been confirmed.

According to Centers for Disease Control and Prevention National Center for Health Statistics (NCHS) the prevalence of obesity was 36.5% in United States among adults during 2011-2014. The prevalence of obesity among middle aged adults (40-59 years) was the highest (40.2%) among all age groups, in both genders (42.1% for females and 38.3% for males).^16^

In a study conducted in European countries, the prevalence of obesity in females ranged between 6.2 to 36.5 % and males between 4 to 28.3% among different countries.^1^ The prevalence of obesity in our country was 36% according to TURDEP-II study. Between TURDEP-I and TURDEP-II surveys, average BMI increased from 26.6 to 28.6 kg/m^2^ and average waist from 87.2 to 94.5 cm over 12 years in Turkey. The rate of increase for abdominal obesity was 35% compared to TURDEP-I.^9^

In our present study, the mean age of all subjects was 46 years. Their mean BMI was 28.1 kg/m^2^ with a mean WaC of 91.3 cm, HC of 104.7 cm, WHR of 0.87. Similar to data reported in the literature, in our present study, we found a high prevalence of obesity in Bursa as 32.2% (37.8% in females and 23.3% in males) according to BMI. Abdominal obesity prevalences were high both with WaC-IDF and WaC-WHO criterias; 63.2% (69.7% in females, 52.9% in males) and 44.4% (53.6% for females, 29.8% for males) respectively. In our present study, females were more obese compared to males both generally and abdominally.

Gender, age, marital status, previous obesity history, education level, accompanying illnesses like diabetes, hypertension, dyslipidemia, atherosclerotic heart disease, hypothyroidism, medications and smoking are known to be the risk factors of obesity.^1^,^2^ In our previous study conducted in 2005, sedentary life style and dyslipidemia in males, being unemployed, having lower level of education and having hypertension in females and familial obesity in both genders were found to be related to increased obesity risk.^10^ In our present study, age, gender, marital status, previous obesity history, hypertension, dyslipidemia, smoking, hypothyroidism and antidiabetic drug usage in diabetics were found to be significant risk factors for obesity development.

The underlying cause of the metabolic syndrome is still debatable. Both general and abdominal obesities contribute to hypertension, dyslipidemia and hyperglycemia and are independently associated with higher cardiovascular disease risk.^16^ The risk of serious health consequences in the form of components of metabolic syndrome has been shown to rise with an increase in BMI, but an excess of body fat in the abdomen is more indicative of the metabolic syndrome profile than BMI.^17^,^18^ In the San Antonio Heart Study, the baseline WaC of the subjects who did not progress to type 2 diabetes was 88.7 cm while who developed type 2 diabetes in 7 years of follow up was 98 cms. In this study WaC was found to be the strongest predictor of type 2 diabetes. In the same study BMI, fasting insulin and triglyceride levels predicted the development of hypertension indicating the importance of overall adiposity^19^ In Nurses Health Study a WaC of 96.5 cm or more was associated with a relative risk of 3.06 for coronary heart disease.^20^

## CONCLUSION

Our results indicated that, both general and abdominal obesities were much more prominent in female gender. All three criterias indicating abdominal obesity namely; WHR, WaC-IDF and WaC-WHO could be preferred for risk determination of metabolic syndrome components. Age, gender, marital status, previous obesity history, hypertension, dyslipidemia, smoking, hypothyroidism and antidiabetic drug usage in diabetic subjects were found to be significant risk factors for obesity development.

Since aging and gender are unmodifyable risk factors, life style changes should be encouraged in the population and preventive measures should be taken by the health authorities to prevent obesity.
